# Copy number variants reveal divergent genetic and diagnostic cortical signatures across psychiatric disorders

**DOI:** 10.21203/rs.3.rs-9246968/v1

**Published:** 2026-05-06

**Authors:** Kuldeep Kumar, Zhijie Liao, Jakub Kopal, Clara Moreau, Christopher Ching, Claudia Modenato, Will Snyder, Sayeh Kazem, Charles-Olivier Martin, Anne-Marie Belanger, Valerie Fontaine, Khadije Jizi, Guillaume Huguet, Rune Boen, Leila Kushan, Ana Silva, Marianne van den Bree, David Linden, Michael Owen, Jeremy Hall, Sarah Lippé, Guillaume Dumas, Bodgan Draganski, Laura Almasy, Sophia Thomopoulos, Neda Jahanshad, Ida Sønderby, Ole Andreassen, David Glahn, Armin Raznahan, Carrie Bearden, Tomas Paus, Paul Thompson, Sebastien Jacquemont

**Affiliations:** Centre de recherche CHU Sainte-Justine and University of Montréal; University of Montreal; Centre for Precision Psychiatry, Division of Mental Health and Addiction, Oslo University Hospital & Institute of Clinical Medicine, University of Oslo; Centre de recherche CHU Sainte Justine, University of Montreal; Imaging Genetics Center, Mark and Mary Stevens Neuroimaging and Informatics Institute, Keck School of Medicine, University of Southern California; Centre Hospitalier Universitaire Vaudois and University of Lausanne; Section on Developmental Neurogenomics, Human Genetics Branch, NIMH, NIH, and Department of Psychiatry, University of Cambridge; Univeristy of Montreal; Centre de recherche CHU Sainte-Justine and University of Montreal; Centre de recherche CHU Sainte-Justine and University of Montreal; University of Montreal; Centre Hospitalier Universitaire Sainte-Justine Research Center; UCLA; University of California Los Angeles; Center for Magnetic Resonance Research, Department of Radiology, University of Minnesota; Cardiff University; Maastricht University; Cardiff University; Cardiff University; University of Montreal; Centre de recherche CHU Sainte-Justine and University of Montreal, and Mila, Quebec Artificial Intelligence Institute; Lausanne University Hospital (CHUV) and University of Lausanne (UNIL),; Children’s Hospital of Philadelphia; University of Southern California; University of Southern California; University of Oslo; Oslo University Hospital & Institute of Clinical Medicine, University of Oslo; Department of Psychiatry, Boston Children’s Hospital and Harvard Medical School, Boston, MA 02115;Harvard Medical School, Boston, MA 02115; National Institute of Mental Health; UCLA; University of Montreal; University of Southern California, Los Angeles; Université de Montréal

## Abstract

Structural variants, including copy number variants (CNVs), confer substantial risk for neurodevelopmental and psychiatric disorders (NPDs), yet whether their cortical effects relate to those observed in the psychiatric conditions they predispose to remains unclear. Here, we present the first systematic comparison of cortical phenotypes across 18 NPD-associated CNVs and aneuploidies, disorder-associated common variants, and 8 psychiatric disorders. Rare CNVs preferentially affected total surface area (SA), with 11-fold larger effects than psychiatric diagnoses, while NPDs preferentially affected mean cortical thickness (CT), with most CT effects observed in medicated subgroups, suggesting non-genetic contributions. NPD-associated common variants showed enrichment in SA but not CT associations. Regionally, both rare and common genetic variants showed larger effects in sensorimotor regions, aligning with the sensorimotor-to-association cortical gradient as well as regional heritability estimates. In contrast, psychiatric diagnoses showed larger effects in association regions. Individual NPD-associated variants were evenly split between those increasing and decreasing surface area. This heterogeneity likely explains why aggregating variants using polygenic scores shows only weak associations with SA. Overall, cortical signatures of psychiatric diagnoses diverge from those associated with genetic risk. Genetic variants preferentially impact SA and sensorimotor regions through early developmental mechanisms, while psychiatric diagnoses are associated with CT and association regions likely reflecting medication, illness chronicity, and environmental factors.

## Introduction

Structural variants, including rare copy number variants (CNVs) and sex chromosome aneuploidies (SCA), alter gene dosage and confer particularly elevated risk for neurodevelopmental and psychiatric disorders (NPDs), with hazard ratios ranging from 2 to over 10 ^1–4^ for NPDs. On the other end of the allele frequency distribution, genome-wide association studies (GWAS) have identified hundreds of common variants associated with conditions including schizophrenia ^5^, bipolar disorder^6^, attention deficit hyperactivity disorder^7^, and depression^8^. Together, both rare and common genetic variants contribute substantially to the heritable architecture of NPDs ^2–4,9–11^. In parallel, large-scale neuroimaging studies, particularly through the ENIGMA consortium^12^, have characterized cortical phenotypes across multiple psychiatric conditions, revealing both disorder-specific and shared patterns of cortical thickness reductions and comparatively modest surface area alterations^13–16^. A central assumption in psychiatric neuroimaging studies is that the cortical phenotypes observed in psychiatric conditions reflect, in part, the effect of genetic variants that increase psychiatric risk. However, case-control neuroimaging studies conflate genetic liability, environmental exposures, medication effects, and consequences of chronic illness, making it difficult to distinguish the cortical signature of genetic risk from non-genetic factors.

A fundamental question therefore remains: are the cortical differences associated with genetic risk variants related to the psychiatric risk they confer? Conversely, do the cortical differences observed in psychiatric disorders reflect their genetic contributions? Convergence would support a genetic interpretation, while divergence would implicate non-genetic factors. Addressing this question requires comparing cortical phenotypes across genetic variants and psychiatric disorders. Previous cross-disorder studies compared cortical thickness across psychiatric conditions^17–20^, but did not incorporate genetic variant data. Neuroimaging studies of individual CNVs have demonstrated robust cortical differences with larger effect sizes than psychiatric conditions^21–23^. However, no study has systematically compared cortical phenotypes across multiple NPD-associated CNVs, common variants, and psychiatric diagnoses.

Cortical thickness (CT) and surface area (SA) are linked to distinct neurobiological processes with largely non-overlapping genetic architectures^24,25^. CT reflects processes including dendritic arborization and myelination continuing into adulthood^26,27^. SA reflects tangential expansion of cortical progenitors during early development and remains stable thereafter^26,28^. These distinct developmental trajectories make CT and SA complementary probes for dissecting genetic versus non-genetic contributions. At the common variant level, prior studies investigating genetic overlap ^24,29–31^ between NPDs and cortical phenotypes have reported weak genetic correlations^24^, but these analyses did not distinguish between CT and SA in the context of cross-variant comparisons.

Here, we systematically characterize cortical phenotypes across 18 NPD-associated CNVs and aneuploidies, constituting the largest cross-variant neuroimaging analysis of structural variants to date, and directly compare them with cortical phenotypes across psychiatric diagnoses. Specifically, we assessed global and regional measures of CT and SA for the following three categories: i) 8 psychiatric disorders, including medication subgroups; ii) 18 NPD-associated CNVs and aneuploidies; and iii) genome-wide significant common variants (SNPs) associated with NPDs. Variants altering gene dosage of 1 or more genes (CNVs and aneuploidies) were selected based on previously established association with one or more psychiatric disorders ^1–4,21,23,32–35^. By characterizing the cortical consequences of structural variants alongside diagnostic and common-variant phenotypes, we reveal a striking dissociation between cortical phenotypes associated with genetic risk, both rare and common, and those associated with psychiatric diagnoses, with implications for the interpretation of case-control psychiatric neuroimaging studies.

## Results

### Structural variants preferentially affect surface area with larger effects than psychiatric diagnoses

We computed effect sizes (Cohen’s d) for cortical phenotypes across 11 CNVs and aneuploidies from individual-level data, supplemented by published meta-analytic estimates for 7 additional variants (Table 1, Supplementary Table 1). Across these 18 structural variants, effects on total SA were consistently larger than on mean CT (paired comparison, FDR q=2E-2, **Supplementary Figures 1A,2A**). Effect sizes (Cohen’s *d*) on total SA were 11-fold larger for rare genetic variants compared with NPDs (Wilcoxon rank-sum test, FDR q=8E-4, Figures 2A-B). This difference in effect sizes was less pronounced for mean CT (4-fold, Wilcoxon rank-sum test, FDR q=7E-3). To assess preferential impact, we computed the ratio of absolute effect sizes for CT and SA in each study. Rare genetic variants showed preferential total SA effects compared to mean CT (median ratio=0.6, one-sample Wilcoxon against 1, FDR q=3E-2, Figure 2C, **Supplementary Figures 1A-B,2A-B**). NPDs showed the opposite pattern with preferential mean CT effects (median ratio=2.1, FDR q=3E-2). This was consistent across deletions, duplications, and aneuploidies (**Supplementary Figure 3**).

Among clinical high-risk individuals, those later developing psychosis (CHR-PS+) showed a CT/SA ratio of 2.9, contrasting with 1.2 for non-converters. The only NPDs showing preferential SA effects were ADHD and conduct disorder, both with childhood onset, but this did not generalize to ASD.

We next tested whether the preferential SA effects observed for rare variants extended to common genetic variants associated with NPDs. We first examined genetic correlations between NPDs and global cortical metrics using summary statistics from Grasby et al. (2020). We observed weak genetic overlap with SA only, where ADHD, BD, and MDD showed significant correlations (FDR q<0.1; **Supplementary Figure 4**). To capture overlap beyond global correlations, we tested whether NPD-associated single-nucleotide polymorphisms (SNPs) were enriched for associations with cortical metrics. We ranked NPD-associated SNPs by their cortical GWAS associations. NPD SNPs showed significant enrichment (above-median ranking) in SA associations for ADHD, BD, and MDD (permutation FDR q<0.0001; **Supplementary Figure 5, Supplementary Table 5**). None showed CT enrichment. Together, these findings indicate that both rare and common NPD-associated variants preferentially associate with total SA rather than mean CT.

### Cortical thickness effects in psychiatric diagnoses reflect non-genetic factors rather than structural variant contributions

We asked if the preferential association of NPDs with mean CT compared to total SA could be in part influenced by non-genetic factors rather than structural variant contributions. To do so, we first assessed NPD medication subgroups (also a proxy for disorder severity ^36^, **Supplementary Table 3**). Effect sizes on mean CT were 8-fold larger in medicated compared to unmedicated subgroups (FDR q=4E-2, Figure 2D-E). Total SA did not differ by medication status. Medicated subgroups showed preferential CT effects (ratio>1), while unmedicated subgroups showed balanced effects (Figure 2F, **Supplementary Figure 1C-D,2C-D**). We acknowledge that medication status may proxy for disorder severity, illness duration, or other clinical factors. Nevertheless, the selective sensitivity of CT, but not SA, to the medication subgroup is consistent with CT reflecting factors that vary with clinical state rather than genetic liability.

Second, using summary statistics from the ENIGMA relatives study ^37^, we examined cortical differences in first-degree relatives of individuals with BD or SCZ. Individuals with BD or SCZ diagnoses showed significantly reduced mean CT compared to controls, while their unaffected first-degree relatives did not show any significant associations with CT (**Supplementary Figure 6**). Neither group showed SA differences. This pattern suggests that CT differences may emerge with, or following, disorder onset, rather than reflecting genetic liability shared by first-degree relatives.

Third, because SA expansion occurs early in life, we asked if SA phenotypes were easier to detect in pediatric NPD groups. This was not the case, and age-stratified analyses in NPDs were unable to detect a clear shift toward associations with SA (**Supplementary Figure 7**), though power limitations and heterogeneity across disorders constrain interpretation.

### Structural variants preferentially affect sensorimotor cortex, diverging from psychiatric diagnostic patterns

We asked if the preferential effects on total SA for genetic variants and mean CT for psychiatric diagnoses were uniformly distributed across the cerebral cortex or localized to specific cortical regions. To do so, we computed the regional cortical effect size maps for 11 CNVs and compared them to regional maps of the 8 NPDs examined above, stratified by age groups and medication **Supplementary Tables 1–3**).

Significant associations with regional SA or CT (at least one ROI) were observed for 11 and 7 out of 18 NPD groups, respectively, and for all 11 CNVs (**Supplementary Figure 8**). We did not detect significant associations for un-medicated sub-groups; as such, the medication sub-groups were excluded from the regional analyses below. Following previous cross-disorder analyses ^17,18,38^, we computed latent dimensions of cortical regional phenotypes using principal component analysis across effect size profiles (Figures 3–4 and **Supplementary Table 6**). The first principal component explained more than a quarter of the variance of regional beta-estimates across CNVs and NPDs, for both CT and SA.

To contextualize these latent regional cortical profiles, we tested their similarities with the well-established cortical gradient (**Methods**), which ranks regions from primary sensory-motor cortices to higher-order association cortices^39,40^. NPD-associated rare genetic variants showed higher loadings in sensorimotor regions compared to association regions (negative correlations with the cortical gradient, Figure 5, and **Supplementary Tables 6,7**). NPDs maps showed the opposite correlations with the cortical gradient, highlighting higher loadings in association regions. The CNVs and NPDs latent dimensions showed negative pairwise correlations (SA: r=−0.38, p-spin < 0.05; CT: r=−0.32, not significant; **Supplementary Figure 9**).

We then asked if our rare variant findings were generalizable to the broader genetic contribution to cortical structure. Towards this, we examined both twin and SNP heritability estimates for regional CT and SA ^24^. Heritability was up to 2-fold higher in sensorimotor regions compared to association regions (**Supplementary Figure 10,** negative correlations with the cortical gradient, r=−0.47 to −0.75, p-spin<0.05, Figure 5). Finally, we assessed the regional profiles of beta estimates from SA GWAS for the NPD-associated common variant (SNPs), to estimate the latent dimension of NPD-associated SNP profiles (**Supplementary Figure 11**). The PC1 for SA explained 24% of variance, and SA-SNPs showed higher effects in sensorimotor regions (r=−0.49, p-spin<0.05) and correlated positively with PC1 for CNVs (SA, r=0.78, p-spin<0.05). The alignment between rare variant effects, common variant effects, and heritability estimates suggests that the sensorimotor preference reflects elemental aspects of how genetic variation influences cortical development, rather than a peculiarity of selected CNVs.

These findings were robust to sensitivity analyses using three alternative consensus cortical maps: mean absolute effect size, percentage of significance, and variance; as well as leave-one-out analyses across CNVs and NPDs (**Supplementary Figures 9,12,13**, and **Supplementary Tables 6,7**). All three showed sensorimotor cortices preference for genetic variants and association cortices preference for NPDs.

### Opposing genetic effects on surface area resolve the polygenic paradox

The preceding analyses reveal a paradox: individual genetic risk variants, both rare CNVs and common SNPs, produce preferential surface area associations with global metric and sensorimotor regions, yet psychiatric disorders show negligible surface area associations. We provide three converging lines of evidence to explain this discrepancy. First, polygenic risk scores (PRS) for BD and SCZ showed no association with total SA in 31,000 UK Biobank participants of European ancestry (**Supplementary Figure 14, Supplementary Tables 8,9**), despite individual BD-associated SNPs showing significant SA enrichment (**Supplementary Figure 5**). To investigate this null PRS finding, we examined effect sizes on SA for each independent genome-wide significant SNP using cortical GWAS summary statistics ^24^, after harmonizing effect alleles between disorder and cortical GWAS. Approximately half of BD- and SCZ-associated SNPs showed negative beta estimates for total SA (BD: 52%; SCZ: 54%; allele harmonized, **Supplementary Figure 14**), with the remainder showing positive effects. A permutation-based variance inflation test confirmed that these disorder-associated SNPs carry individually meaningful SA effects: their cortical effect sizes were significantly more variable than those of minor allele frequency (MAF) matched random SNPs (SCZ: variance ratio = 1.32, p=0.037; BD: variance ratio = 1.23, p=0.089; 10,000 permutations, **Supplementary Table 10**), despite the near-zero mean. Simulations using empirical SNP effect sizes demonstrated that under the observed distribution of positive and negative cortical effects, the aggregate PRS–SA association (R^2^ ≈ 10^−5^) is over two orders of magnitude weaker than when all effects are forced to align in the same direction (R^2^ ≈ 6 × 10^−3^), without changing their magnitudes (10,000 simulations; **Supplementary Table 11**). Thus, cancellation of opposing effects, not the absence of individual SNP effects, accounts for the null PRS–SA finding.

Second, this balanced proportion of negative and positive effects on regional SA extended to rare variants (CNVs). Nearly half of the CNVs loaded positively while the other half loaded negatively on the first regional SA latent dimension (Figure 3D). NPD-associated SNPs showed a similar balanced proportion of negative and positive loadings (**Supplementary Figure 11B**). Third, consistent with polygenic cancellation, unaffected first-degree relatives of a proband with a psychiatric diagnosis, a proxy for multifactorial (including polygenic) liability, also showed weak associations with SA (similar to probands, **Supplementary Figure 6**). Together, these findings resolve the apparent contradiction between i) high SA heritability, ii) clear individual variant effects on SA, and iii) null associations between NPD-PRS and SA.

## Discussion

This study provides the first systematic characterization of cortical phenotypic consequences across multiple NPD-associated structural variants (CNVs and aneuploidies), revealing a striking dissociation with cortical signatures of the psychiatric conditions these variants predispose to. Genetic variants associated with NPDs, both rare CNVs and common SNPs, preferentially affected surface area, while psychiatric conditions preferentially affected cortical thickness. This dissociation extended to regional patterns: genetic variants showed larger effects in sensorimotor cortical regions, while psychiatric diagnoses showed larger effects in association regions. We further demonstrate that individual genetic variants produce clear SA effects that are heterogeneous and cancel out when genetic variants are aggregated into polygenic scores. These heterogeneous effects may also explain why the association between NPDs (polygenic conditions) and cortical SA remains difficult to detect at the group level.

For the structural variant field, our findings demonstrate that systematic cross-variant neuroimaging analyses reveal shared properties of CNV effects on brain structure, including preferential SA and sensorimotor impact, that would be invisible when studying individual variants in isolation ^21,23,33,34,41^. Notably, these properties extend to NPD-associated common variants, indicating a consistent genetic architecture of cortical effects across the allele frequency spectrum. A distinct but complementary finding is that individual structural and common variants show both positive and negative effects on SA, resolving why these clear individual-variant effects vanish in aggregate genetic analyses. Cortical surface area in sensorimotor regions may therefore represent a more genetically interpretable and sensitive endophenotype for structural variant effects than cortical thickness, which appears more sensitive to non-genetic factors. These results underscore the value of phenotyping structural variants through neuroimaging to understand how altered gene dosage shapes cortical development.

The preferential association between genetic variants and total SA aligns with the higher heritability estimates for total SA compared to mean CT^24–27,42^. NPD-associated variants impacting SA suggest that they operate through early mechanisms of cortical expansion^24,27,43^. This SA preference is consistent across diverse structural variants, including deletions, duplications, and aneuploidies, and aligns with recent evidence that CNV deletions and duplications across the genome reduce surface area by disrupting the proliferation of neural progenitor cells during fetal cortical development^43^. The preferential association between mean CT and psychiatric diagnoses may reflect non-genetic factors. Medicated subgroups showed larger CT but not SA effects, consistent with randomized trial evidence of medication-induced CT changes ^44^. We acknowledge that medication status may co-vary with disorder severity, illness duration, or other clinical variables. Disentangling medication effects from illness severity requires longitudinal and randomized designs beyond the scope of this cross-sectional analysis.

At the regional level, structural and common genetic variants preferentially affected sensorimotor regions, which develop earlier ^39^ and show higher heritability ^24–27,42^ compared to association regions. Psychiatric diagnoses were preferentially associated with association cortices, consistent with prior cross-disorder studies reporting frontotemporal thickness reductions ^17,18^. Notably, even within a single structural variant, the 22q11.2 deletion, carriers who develop psychosis show focal CT reductions in association regions compared to non-psychotic carriers, consistent with diagnosis-related CT effects being layered on top of the genetic signature^45^. Association regions with protracted development may be more vulnerable to environmental perturbations and illness-related plasticity, while sensorimotor regions may reflect earlier effects of genetic liability.

A central puzzle in psychiatric and neuroimaging genetics has been the weak genetic correlations between NPDs and cortical structure, despite substantial heritability of both and the assumption that NPD risk conferred by genetic variants is mechanistically related to the effects of the same variants on brain structure ^30^. Previous studies have demonstrated that genetic overlap (of common variants) between two traits may be under-estimated by genetic correlation values due to the heterogeneous effects of genetic variants ^29,46^. In line with these studies, we show that rare and common genetic variants increasing risk for NPDs do associate with cortical structure, particularly SA, suggesting a higher genetic overlap despite weak genetic correlations^24,30^. However, we show that their effects are heterogeneous, with roughly equal numbers of NPD-risk variants increasing versus decreasing SA. When genetic-risk variants are aggregated into additive polygenic risk scores for NPDs or in psychiatric neuroimaging case-control group analysis, these heterogeneous/opposing effects on SA are canceled, producing null or weak associations.

Our findings have several implications for psychiatric neuroimaging research. The CT and association-region effects predominant in psychiatric diagnoses may largely reflect consequences of illness, including medication, chronicity, comorbidity, and lifestyle factors, rather than the genetic variants that increase disorder risk. We note that this does not diminish the importance of such findings for understanding disease burden or treatment effects, but it reframes their interpretation. Second, additive polygenic models have limitations for imaging outcomes; methods separating positive and negative effects may improve prediction. Third, cross-sectional case-control designs conflate genetic and non-genetic contributions; longitudinal designs beginning before illness onset are needed to disentangle genetic and non-genetic contributions.

Several limitations of our study warrant consideration. ENIGMA summary statistics were derived from heterogeneous studies with varying protocols, age ranges, and clinical characterization. While ENIGMA’s harmonization procedures mitigate protocol differences, residual heterogeneity may affect comparisons across disorders. Medication subgroup analyses are constrained by the information available in ENIGMA publications; we could not examine dose-response or duration. Third, while genetic risk variants have strong effects on SA, with substantial heterogeneity, our study design does not allow us to distinguish whether SA phenotypes are related to mechanisms associated with increasing risk for NPDs or merely reflect the highly pleiotropic nature of these variants ^47,48^.

Overall, genetic variants preferentially affect surface area and sensorimotor regions, consistent with higher heritability and early developmental processes involved in these regions and phenotypes. Psychiatric diagnoses are preferentially associated with cortical thickness and association regions, with sensitivity to medication status suggesting non-genetic contributions. NPD-associated risk variants were evenly split between those increasing and decreasing cortical surface area, likely explaining why aggregating risk variants for NPDs using polygenic scores only yields weak correlations with this cortical phenotype. These findings suggest that case-control differences in psychiatric neuroimaging may reflect other factors, including consequences of illness rather than its genetic contributions, with implications for interpretation of case-control findings. By systematically characterizing cortical consequences across multiple structural variants, this study provides a framework for understanding how gene dosage alterations shape brain phenotypes and why these effects remain largely undetectable in aggregate genetic or diagnostic analyses.

## Methods

### Participants

**Figure 1:**
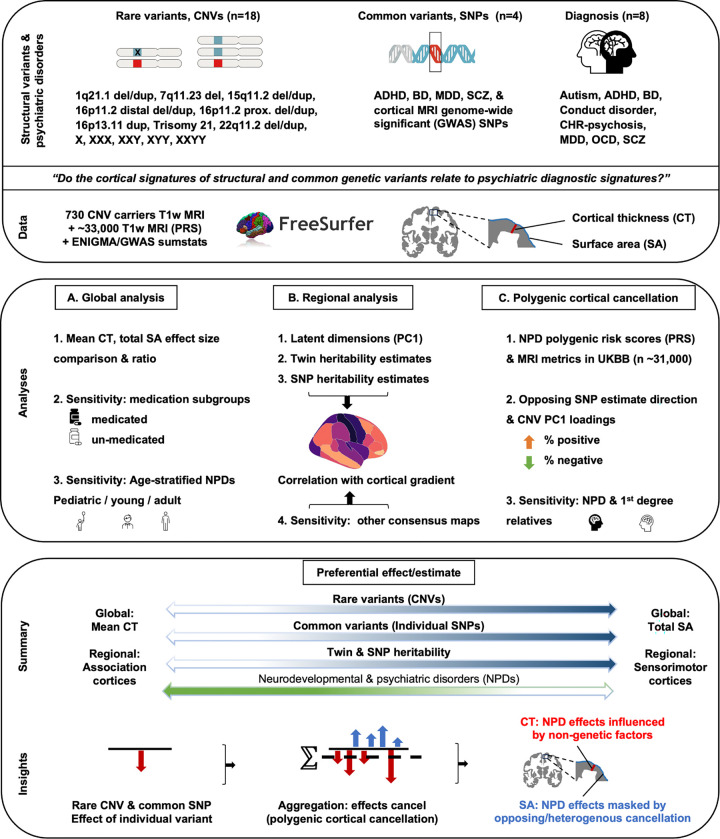
Study overview. We compared cortical thickness (CT) and surface area (SA) effect sizes across three categories of NPD-related data in ~33,000 individuals: i) 18 NPD-associated rare structural variants (CNVs and aneuploidies; 730 carriers and 870 matched controls); ii) genome-wide significant common variants (SNPs) from PGC GWAS for 4 NPDs, cross-referenced with ENIGMA cortical GWAS; and iii) 8 psychiatric disorders from ENIGMA consortium summary statistics, including medication and age-stratified subgroups. CT and SA were extracted using FreeSurfer. **A**) Global analysis: CT and SA effect sizes were compared across categories, with sensitivity analyses for medication subgroups and age-stratified samples. **B**) Regional analysis: latent dimensions of regional cortical differences (PCA) were correlated with the sensorimotor-to-association cortical gradient and with twin and SNP heritability estimates; alternative consensus maps (mean absolute effect size, percentage of significance, and variance) served as sensitivity analyses. **C**) Polygenic cortical cancellation: polygenic risk scores (PRS) for NPDs were tested against cortical metrics in UK Biobank (N~31,000), and directionality of individual SNP and CNV effects was assessed; first-degree relatives of probands served as a sensitivity analysis. Summary: Genetic variants (both rare and common) preferentially affect total SA and sensorimotor regions, aligning with heritability estimates, while NPD diagnoses preferentially affect mean CT and association cortices. Insights: Individual genetic variants produce bidirectional cortical effects (~50% positive, ~50% negative for SA), which cancel in additive polygenic models, explaining weak NPD effect sizes for SA. CT effects in NPDs appear driven by non-genetic factors (medication, chronicity), while SA effects are masked by bidirectional cancellation. Brain and cortex maps were generated using the ggseg package in R(50). Common and rare variant illustrations are from the NIAID NIH BIOART Source (https://bioart.niaid.nih.gov/bioart/170 and https://bioart.niaid.nih.gov/bioart/204)

**Figure 2:**
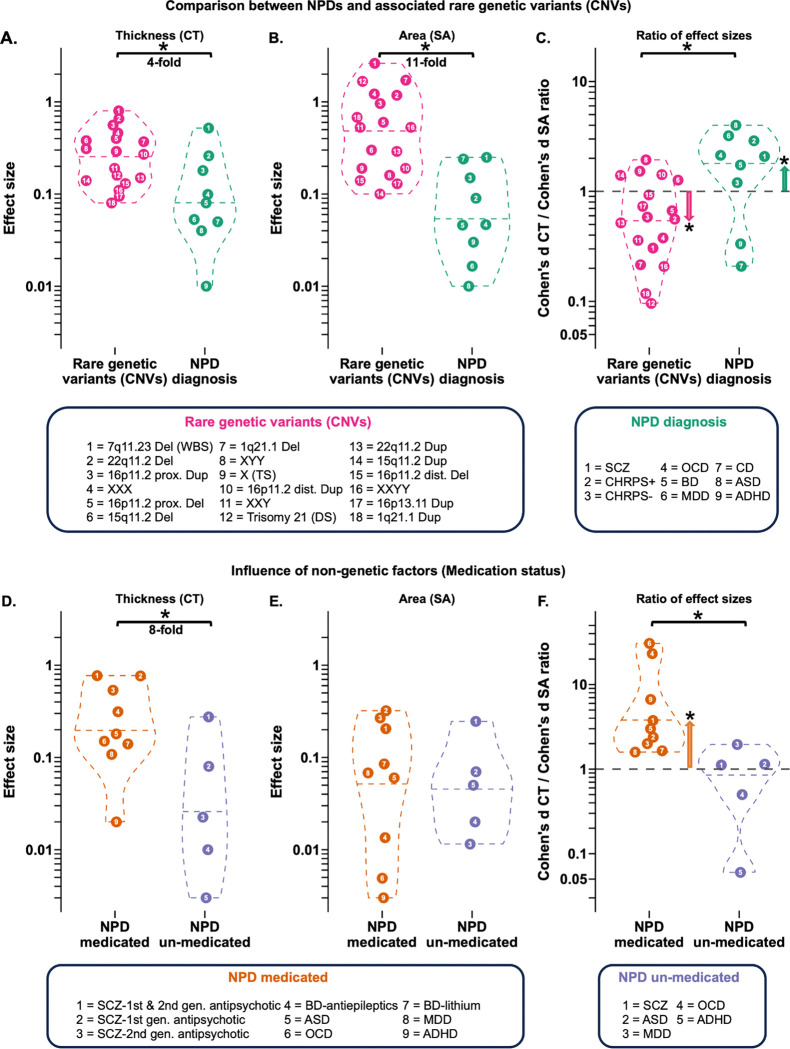
Global cortical differences across genetic variants and psychiatric diagnoses. **A-C**) Comparing cortical phenotypes between neurodevelopmental and psychiatric disorders (NPDs), and rare genetic variants (CNVs) associated with NPDs, for **A**) mean cortical thickness (CT) effect sizes; **B**) total surface area (SA) effect sizes; **C**) Ratio of Cohen’s d CT and Cohen’s d SA (effect sizes). **D-F**) Comparing sub-groups with and without medications across NPDs for **D**) mean cortical thickness (CT) effect sizes; **E**) total surface area (SA) effect sizes; **F**) Ratio of Cohen’s d CT and Cohen’s d SA. Case-control differences were adjusted for age, sex, and site. Effect sizes correspond to absolute Cohen’s d values. *: FDR significant (q<0.05), across all pairs of comparisons. For panel C and F, * and arrows denote the direction of the median ratio shift from 1 (FDR q<0.05). Y-axis: Absolute effect sizes, and effect size ratios, plotted on a log10 scale. Abbreviations: Abs=absolute; ADHD=attention deficit hyperactivity disorder; ASD=autism spectrum disorder; BD=bipolar disorder; CD: conduct disorder; CHR-PS: clinical high risk for psychosis; CHR-PS−: CHR who did not develop a psychotic disorder; CHR-PS+: CHR who later developed a psychotic disorder; CNV=copy number variant; CT=cortical thickness; Del=deletion; Dup=duplication; MDD=major depressive disorder; NPD=neurodevelopmental and psychiatric disorders; OCD=obsessive-compulsive disorder; prox.=proximal; SA=surface area; SCZ=schizophrenia; TS=Turner syndrome; WBS=Williams-Beuren syndrome;

**Figure 3:**
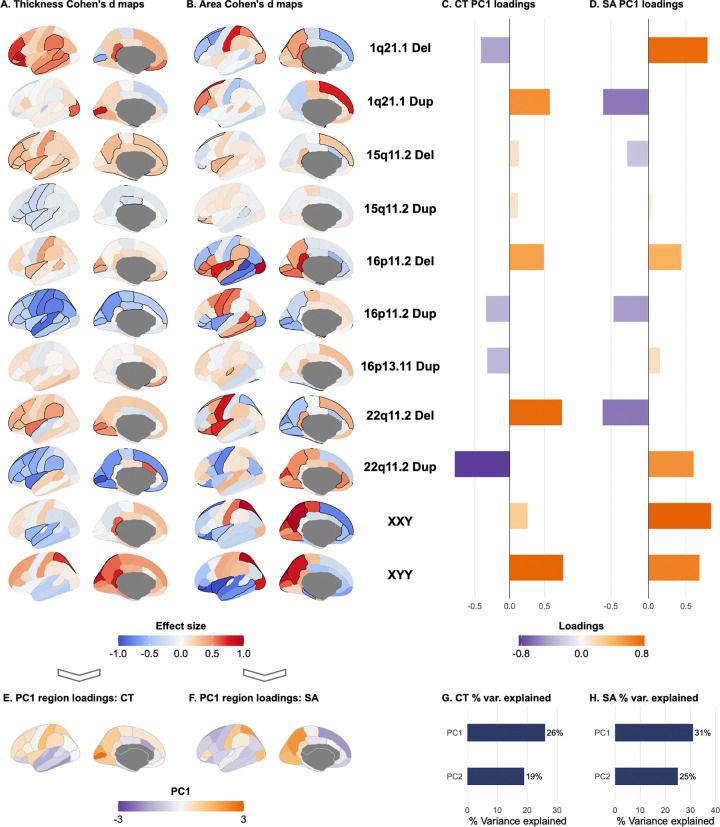
Regional cortical alterations across rare genetic variants. Cohen’s *d* maps for regional cortical alterations for 11 rare genetic variants (CNVs, including aneuploidies): **A**) cortical thickness; **B**) surface area. Case-control differences were calculated after adjusting for age, sex, and site (and total SA for SA). FDR (q<0.05) significant regions are shown in black boundaries. **C-H**) Principal component analysis across regional cortical differences. PC1 variable loadings for **C**) CT and **D**) SA. Latent dimension map: the first principal component from the principal component analysis (PCA) across **E**) CT and **F**) SA alterations for CNVs. The variance explained by the first two principal components (PC1 and PC2) for **G**) CT, and **H**) SA. Abbreviations. CNV=copy number variants; Corr.=correlation; CT: cortical thickness; Del=deletions; Dup=duplications; PC: principal component; SA=surface area; % var. explained = percentage of variance explained.

**Figure 4:**
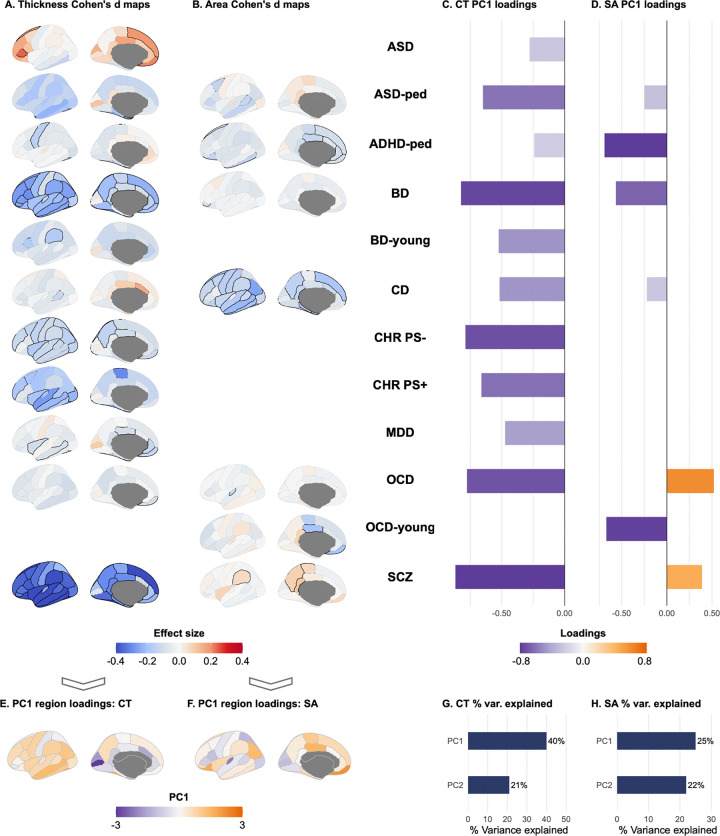
Regional cortical alterations across neurodevelopmental and psychiatric disorders. Cohen’s *d* maps for regional cortical alterations across neurodevelopmental and psychiatric disorders (NPDs) for **A**) cortical thickness (CT); **B**) surface area (SA). Case-control differences were calculated after adjusting for age, sex, and site (and total SA for SA). FDR (q<0.05) significant regions are shown by black boundaries. Only SA and CT effect sizes maps with FDR significant associations for at least one ROI are shown. **C-H**) Principal component analysis across regional cortical differences. PC1 variable loadings for **C**) CT and **D**) SA. Latent dimension map: the first principal component from the principal component analysis (PCA) across **E**) CT and **F**) SA alterations for NPDs. The variance explained by the first two principal components (PC1 and PC2) for **G**) CT, and **H**) SA. Abbreviations. ADHD=attention deficit hyperactivity disorder; ASD=autism spectrum disorder; BD=bipolar disorder; CD: conduct disorder; CHR-PS: clinical high risk for psychosis; CHR-PS−: CHR who did not develop a psychotic disorder; CHR-PS+: CHR who later developed a psychotic disorder; CT=cortical thickness; MDD=major depressive disorder; NPD=neurodevelopmental and psychiatric disorders; OCD=obsessive-compulsive disorder; PC: principal component; SA=surface area; SCZ=schizophrenia; % var. explained = percentage of variance explained.

**Figure 5:**
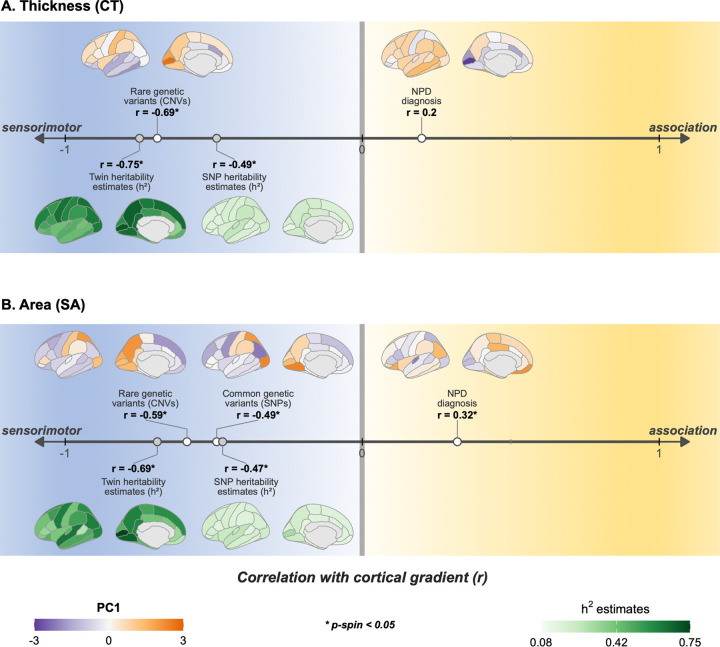
Correlation between the cortical gradient and latent dimensions of regional cortical differences. Correlation between the cortical gradient (Pearson’s r) and latent dimension (PC1) of regional differences / regional heritability estimates for **A**) cortical thickness (CT) and **B**) surface area (SA). Latent dimension (PC1) profiles were computed for effect sizes across rare genetic variants (CNVs), common genetic variants (SNPs), and NPDs for cortical thickness and surface area across 34 Desikan cortical regions. Regional profiles of twin and SNP heritability estimates for cortical thickness and surface area across 34 Desikan cortical regions from Grasby et al. ^24^. The regional surface area estimates are adjusted for Total SA. Abbreviations, CT=cortical thickness; h2: heritability; NPD=neurodevelopmental and psychiatric disorders; PC: principal component; SA=surface area; SNP: single nucleotide polymorphism; r= Pearson correlation; *:spin-permutation significant, p-spin < 0.05.

We analyzed CT and SA integrating individual-level neuroimaging data from approximately 33,000 participants (730 CNV/aneuploidy carriers, 870 matched controls, and 31,413 UK Biobank participants for polygenic risk score analyses), together with published ENIGMA summary statistics for 8 psychiatric disorders (Figure 1, Table 1, **Supplementary Tables 1–4**). ENIGMA summary statistics encompassed approximately 12,600 cases and 19,200 controls across 8 psychiatric disorders (**Supplementary Table 2**), along with medication, age-stratified, and diagnostic subgroups (**Supplementary Table 3**).

#### Rare genetic variant participants

We used individual-level neuroimaging data for carriers of recurrent CNVs or sex chromosome aneuploidies from clinical cohorts and the UK Biobank general population sample. The final sample included 730 carriers of 18 distinct variants and 870 matched controls. Demographic details, coordinates of each of the CNVs, as well as NPD risk, the Hazard ratios (HR) or Odds Ratios (OR) for each CNV, derived from the Danish iPSYCH2015 dataset^1,2^ and Modenato et al. ^21^ are provided in Table 1 and **Supplement Table 1**.

##### Ethics:

Signed consents were obtained by investigators from each cohort for all participants and/or their legal representatives prior to the investigation. This study was approved by the Ethics committee from the CHU Sainte-Justine Hospital.

##### Clinical cohorts:

CNV carriers were recruited following genetic testing referral for neurodevelopmental concerns or as relatives of identified carriers. Contributing cohorts included: 16p11.2 European Consortium, Brain Canada multi-site cohort, UCLA 22q11.2 deletion syndrome cohort, Cardiff University CNV cohort, and NIMH sex chromosome aneuploidy cohort (Detailed in prior publications ^34^). Controls were defined as individuals from the same cohorts not carrying any NPD-associated CNVs at the examined loci.

##### UK Biobank:

Additional CNV carriers were identified in the UK Biobank^49^ general population sample (application 40980) using a validated CNV calling pipeline ^22,41^. UK Biobank controls were participants not carrying any recurrent CNVs selected for this study.

#### Psychiatric disorder and cortical phenotype associated common variants

Genome-wide significant SNPs were extracted from published Psychiatric Genomics Consortium GWAS for ADHD ^7^; BD^6^; MDD ^8^; and SCZ ^5^ (Table 1 and **Supplementary Tables 4,5**). To assess cortical associations of these NPD-associated SNPs, we obtained estimates from the ENIGMA cortical structure GWAS ^24^, which provides effect estimates and association statistics for regional and global CT and SA measures. We also obtained genetic correlation estimates (rg) between NPDs and global cortical metrics from the same source ^24^. This allowed two complementary approaches: (i) genome-wide genetic correlations to assess genetic overlap between NPDs and cortical structure, and (ii) a SNP-level cross-referencing approach testing whether variants identified through psychiatric GWAS show enrichment for cortical structure associations beyond what genome-wide correlations capture.

#### Neurodevelopmental and psychiatric disorder (NPD) participants

In this study, we analyzed published summary statistics for 8 psychiatric disorders from the following published ENIGMA^12^ studies: attention-deficit hyperactivity disorder (ADHD)^13,50^; autism spectrum disorder (ASD)^13,51^; bipolar disorder (BD)^14^; clinical high-risk for psychosis (CHR-PS)^52^; conduct disorder (CD)^53^; major depressive disorder (MDD)^15^; obsessive-compulsive disorder (OCD)^13,54^; and schizophrenia (SCZ)^16^) (**Supplementary Tables 2,3).** Where available, we extracted summary statistics for: (i) medication subgroups (BD: lithium, antiepileptics, atypical antipsychotics; SCZ: first-generation antipsychotics, second-generation antipsychotics, combined); (ii) age-stratified samples (pediatric, adolescent/young adult, adult); and (iii) diagnostic subtypes (CHR-PS converters versus non-converters).

### MRI acquisition and preprocessing

#### Rare genetic variants

T1-weighted volumetric images at 0.8–1mm isotropic resolution were acquired across contributing sites using 1.5T and 3T scanners from multiple vendors (Siemens, GE, Philips). Detailed acquisition parameters for each cohort are provided in Supplementary Information. Visual quality control was performed by two trained raters (C.M., K.K.) following ENIGMA standardized protocols (https://github.com/ENIGMA-git). Sex chromosome aneuploidy samples underwent separate quality control (W.S., A.R.) using equivalent criteria.

#### Neurodevelopmental and psychiatric disorder samples

All contributing ENIGMA working groups followed standardized quality control and FreeSurfer ^55^ processing protocols as described in respective source publications.

#### Cortical metric extraction

FreeSurfer version 5.3.0 was used to extract cortical thickness (CT) and surface area (SA) for 68 regions of the Desikan-Killiany atlas, plus global measures (total surface area, mean cortical thickness). Left and right hemisphere values were averaged for primary analyses to reduce multiple comparisons and because i) some of the ENIGMA summary statistics are only available for averaged metrics; and ii) prior work has shown high bilateral correlations for these metrics in CNV carriers^21,22,41^. UK Biobank samples used for PRS analyses were processed using FreeSurfer 6.0 via the UK Biobank imaging pipeline ^56^.

### Statistical analysis

#### Effect size computation

For rare genetic variants, Cohen’s d effect sizes for carrier-control differences were computed using linear regression models adjusting for age, sex, and site. For regional surface area analyses, total surface area was included as an additional covariate to isolate regional effects from global scaling, consistent with ENIGMA protocols.

For NPDs, Cohen’s d values were extracted directly from published ENIGMA studies. All NPD effect sizes were computed with adjustment for age, sex, and site. Regional surface area analyses in ENIGMA studies included adjustment for total SA or intracranial volume, except for MDD, where such adjusted results were unavailable. Summary statistics from ENIGMA studies used site-covariate approaches. As such, site effects were modeled as covariates in all analyses. Maintaining the same statistical framework across NPD and CNV analyses ensured consistency.

For additional CNV and aneuploidy variants with smaller sample sizes in our cohorts (Turner syndrome, Down syndrome, XXX, XXYY, Williams-Beuren syndrome, 16p11.2 distal deletion/duplication), we utilized previously published meta-analytic effect sizes from the literature ^21^ (**Supplementary Table 1**).

#### Multiple comparison correction

False discovery rate (FDR) correction using the Benjamini-Hochberg procedure was applied within each analysis. Statistical significance threshold was set at FDR-corrected q<0.05.

#### Rationale for cross-variant and cross-disorder analyses

Because many individuals meet criteria for several psychiatric disorders during their lifetime, there have been significant cross-disorder efforts to study the genetic architecture across psychiatric disorders ^9,11^. The same rationale has led neuroimaging consortia to investigate neurobiological processes across psychiatric conditions. Neuroimaging studies of individual CNVs have demonstrated distinct clinical features and brain signatures ^21,22,33,34,41^. However, a cross-disorder, cross-CNV neuroimaging investigation has not yet been conducted.

#### Assessing non-structural variant contributions using medication subgroup analysis

Effect sizes for medicated versus unmedicated NPD subgroups were extracted from published ENIGMA studies where available. We compared effect sizes between medication subgroups using Wilcoxon rank-sum tests and computed CT/SA ratios for each subgroup to assess whether medication status differentially influenced the two metrics.

We acknowledge that medication status in observational studies may proxy for disorder severity, illness duration, treatment resistance, or other clinical factors that co-vary with pharmacological treatment. Causal interpretation of medication effects requires randomized designs beyond the scope of this cross-sectional analysis.

#### Global metric comparisons

We compared absolute Cohen’s d values between CNVs/aneuploidies and NPDs using Wilcoxon rank-sum tests. To assess preferential effects on CT versus SA within each category, we computed the ratio of absolute effect sizes (*Cohen_d_CT / Cohen_d_SA*) for each condition and tested whether median ratios differed from 1 (indicating balanced effects) using one-sample Wilcoxon signed-rank tests. Paired comparisons of CT versus SA effect size distributions within CNV and NPD groups used paired t-tests.

#### Common variant enrichment analysis

To test whether NPD-associated SNPs showed enrichment for cortical structure associations beyond chance, we employed a ranking-based approach. All SNPs with available data in the ENIGMA cortical GWAS were ranked by their p-value association with CT or SA. For each NPD, we identified genome-wide significant SNPs (p<5E-8) and computed the median rank of these SNPs within the cortical GWAS ranking.

Statistical significance was assessed using permutation testing (10,000 permutations), randomly sampling equal numbers of SNPs from the full GWAS and recomputing median ranks to generate a null distribution. Enrichment was defined as median rank significantly above the 50th percentile (i.e., NPD SNPs ranked higher than expected by chance in cortical associations). FDR correction was applied across the four NPDs tested. Reciprocal analyses ranked cortical GWAS significant SNPs within NPD GWAS to assess bidirectional enrichment.

#### Common variant variance inflation test

To test whether disorder-associated SNPs carry individually meaningful effects on cortical surface area, we performed a permutation-based variance inflation test. For each disorder (BD, SCZ), we identified independent genome-wide significant lead SNPs from published GWAS (BD: 261 loci^6^; SCZ: 287 loci^5^) and extracted their total surface area effect sizes from the ENIGMA cortical GWAS summary statistics^24^. Effect alleles were harmonized between the disorder and cortical GWAS; SNPs with unresolvable allele mismatches were excluded, yielding 234 BD and 259 SCZ SNPs. We computed the observed variance of SA betas across disorder-associated SNPs and compared it to a null distribution generated by randomly sampling the same number of SNPs from the full cortical GWAS, matched on minor allele frequency using decile bins (10,000 permutations). The one-sided permutation p-value was computed as the proportion of null variance values equal to or exceeding the observed variance.

#### Regional consensus maps

To characterize consistent regional patterns for the cross-CNVs and cross-NPDs comparison, we followed the approach used in prior cross-disorder studies^17,18,34,38,41^ and ran Principal Component Analysis (PCA). PCA was performed on the matrix of Cohen’s d values (regions × conditions) using the FactoMineR^57^ package. The first principal component (PC1) captures the dominant pattern of covariation across conditions. PC1 loadings were aligned so that regions with higher variance corresponded to positive loadings for interpretability. Only conditions with at least one FDR-significant regional association were included in regional consensus analyses, ensuring patterns were driven by detectable effects. This criterion excluded medication subgroups, which showed no significant regional effects individually.

As a sensitivity analysis, we computed regional consensus maps using three complementary approaches. i) Mean absolute effect size: Mean of absolute *Cohen’s d* values per region across all conditions within each category, identifying regions with consistently large effects. ii) Percentage significance: Proportion of conditions showing FDR-significant effects per region, identifying regions frequently affected regardless of effect magnitude. and iii) Variance: Variance in *Cohen’s d* values per region across conditions, identifying regions of heterogeneous versus homogeneous effects.

#### Cortical gradient analysis

Regional profiles were correlated with the sensorimotor-to-association cortical gradient derived from the first principal component of gene expression across cortical regions ^39,40^. This gradient ranks regions from primary sensory-motor cortices (low values) to higher-order association cortices (high values), capturing the hierarchy of cortical organization related to development, connectivity, and function. Gradient values for 34 left hemisphere Desikan-Killiany regions were obtained using the neuromaps package ^40^.

#### Statistical significance of spatial correlations

Statistical significance of spatial correlations was assessed using spin permutation^58^ testing (1,000 permutations) to account for spatial autocorrelation in cortical data. Spin permutation preserves the spatial structure of the cortex while generating null distributions, providing more conservative and appropriate inference than parametric tests for spatially embedded data.

### Brain map visualizations were generated using *ggseg* ^59^.

#### Twin and SNP Heritability Estimates

Twin heritability and SNP heritability estimates for regional CT and SA were obtained from published ENIGMA genetic architecture studies ^24^. Regional heritability profiles were correlated with the cortical gradient using spin permutation^58^ testing to assess whether genetic determination of cortical structure varies systematically across the cortical hierarchy.

#### Polygenic risk score analysis

Standard polygenic risk scores (PRS) for BD and SCZ were obtained from UK Biobank (data fields 26214, 26275), computed using established methods with optimized p-value thresholds. We restricted analyses to participants of European ancestry (to match GWAS discovery samples) who did not carry any of the recurrent CNVs examined in this study (final n=31,000, **Supplementary Table 8**). Linear regression models tested associations between PRS and cortical metrics, adjusting for age, sex, imaging assessment center, and the first 10 genetic ancestry principal components. For regional SA analyses, total SA was included as an additional covariate. FDR correction was applied across all PRS-cortical associations (**Supplementary Table 9**). To examine the directionality of individual SNP effects, we extracted beta estimates for NPD-associated SNPs from the ENIGMA cortical structure GWAS^24^ and computed the proportion with negative versus positive effects on CT and SA. This analysis tests whether the null PRS associations reflect the true absence of genetic effects or cancellation of opposing individual SNP effects.

#### Polygenic risk score (PRS)–surface area synthetic simulation

To quantify the impact of opposing SNP effects on PRS–surface area associations, we simulated PRS–SA associations under empirical and counterfactual sign distributions. For each disorder, we used the harmonized set of independent lead SNPs, their disorder effect sizes (log-odds ratios), and their SA effect sizes standardized to a per-SD scale by dividing by the phenotype standard deviation estimated from the GWAS summary statistics. For each of 10,000 replicates, genotypes for 31,000 synthetic individuals were drawn from a binomial distribution (n = 2, p = MAF) at each SNP. PRS was computed as the weighted sum of genotypes using disorder effect sizes. The cortical phenotype was constructed as the sum of a genetic component (weighted by standardized cortical betas) and Gaussian noise, calibrated so that the genetic component contributed approximately 1% of total phenotypic variance, consistent with the expected contribution of ~240 genome-wide significant loci. Two scenarios were compared: the empirical scenario, using cortical betas with their original signs, and the concordant scenario, in which all cortical betas were forced to share the sign of the corresponding disorder beta while preserving their magnitudes. R^2^ was computed for each replicate as the squared correlation between PRS and the simulated cortical phenotype.

#### First Degree Relatives analysis

Effect sizes for first-degree relatives (parents and siblings) of BD and SCZ probands were extracted from published ENIGMA relatives study summary statistics^37^. We compared effect sizes between diagnosed probands and their unaffected relatives using descriptive approaches and assessed whether relative effects were significantly different from zero using one-sample t-tests, testing the hypothesis that cortical differences emerge with disorder diagnosis rather than being present in at-risk individuals.

## Supplementary Material

Supplementary Files

This is a list of supplementary files associated with this preprint. Click to download.

• SupplementaryTables.xlsx

• SupplementaryMaterial.docx

## Figures and Tables

**Table 1.** Description of structural variants, psychiatric disorders, and disorder summary statistics.

A. Rare Genetic Variants (CNVs and Aneuploidies)	Type	N (Cases / Ctrls)	HR ADHD	HR BD	HR MDD	OR ASD	OR SCZ
1q21.1 Deletion	CNV	40 / 782	1.93	3.52	2.08	1.56	6
1q21.1 Duplication	CNV	30 / 782	2.65	1.63	1.43	8.03	3
7q11.23 Deletion (WBS)	CNV	44 (meta)	—	—	—	32	—
15q11.2 Deletion	CNV	108 / 782	1.43	0.63	1.17	1.3	1
15q11.2 Duplication	CNV	144 / 782	1.42	1.26	1.07	1.8	1
16p11.2 Deletion (distal)	CNV	15 (meta)	1.26	—	1.04	1.73	4
16p11.2 Duplication (distal)	CNV	18 (meta)	1.57	1.89	1.17	1.15	1
16p11.2 Deletion (proximal)	CNV	82 / 782	0.77	—	0.42	9.5	1
16p11.2 Duplication (proximal)	CNV	75 / 782	2.63	0.53	1.04	11.81	12
16p13.11 Duplication	CNV	50 / 782	2.01	0.8	1.38	1.5	2
Trisomy 21 (Down Syndrome)	Aneuploidy	84 (meta)		—	—	6.83	3.67
22q11.2 Deletion (VCFS)	CNV	68 / 782	1.17	—	1.03	32.37	92
22q11.2 Duplication	CNV	26 / 782	2.61	—	1.02	3.28	0.15
XXX Syndrome	Aneuploidy	35 (meta)	2.67	4.32	2.2	5.6	17.86
XXY (Klinefelter)	Aneuploidy	77 / 53	1.99	1.62	1.88	4	17.86
XYY Syndrome	Aneuploidy	30 / 35	4.45	3.22	2.65	4.6	
XXYY Syndrome	Aneuploidy	25 (meta)		—	—	1.92	
X Monosomy (Turner)	Aneuploidy	55 (meta)	6.15	—	1.62	—	
B. Neurodevelopmental & Psychiatric Disorders	PubMed ID	N (Cases / Ctrls)	Source	Medication subgroups	Subgroups		
ADHD (AttentionDeficit /Hyperactivity Disorder)	32539527	2271 / 5827	ENIGMA	Yes	Ped, Young, Adult		
ASD (Autism Spectrum Disorder)	32539527	1777 / 5827	ENIGMA	Yes	Ped, Young, Adult		
BD (Bipolar Disorder)	28461699	2447 / 4056	ENIGMA	Yes	Young, Adult		
CD (Conduct Disorder)	39025633	1185 / 1253	ENIGMA	No	Young		
CHR-PS (Clinical High Risk for Psychosis)	33950164	1792 / 1377	ENIGMA	No	Converters / Non		
MDD (Major Depressive Disorder)	27137745	2148 / 7957	ENIGMA	Yes	Young, Adult		
OCD (Obsessive Compulsive Disorder)	32539527	2323 / 5827	ENIGMA	Yes	Ped, Young, Adult		
SCZ (Schizophrenia)	29960671	4474 / 5098	ENIGMA	Yes	Adult		
C. Common Variants (GWAS Statistics)	Reference	N (Cases / Ctrls)	Source	N Signif SNPs	SNP heritability		
Attention-deficit/hyperactivity disorder (ADHD)	Demontis et al., 2023	38691 / 186843	PGC	27	14% (1%)		
Bipolar Disorder (BD)	O’Connell et al., 2025	131969 / 2322416	PGC	239	22% (1%)		
Major Depression (MDD)	Meng et al., 2024	258364 / 571252	PGC	180	8.4% (0.07%)		
Schizophrenia (SCZ)	Trubetskoy et al., 2022	76755 / 243649	PGC	259	24% (0.07%)		
Cortical MRI (Mean CT)	Grasby et al., 2020	51,665 (Total)	ENIGMA	6	26% (2%)		
Cortical MRI (Total SA)	Grasby et al., 2020	51,665 (Total)	ENIGMA	20	34% (3%)		

This study integrates data across three domains: **A**) Rare genetic variants, including copy number variants (CNVs) and sex chromosome aneuploidies (SCAs); **B**) Neurodevelopmental and psychiatric disorders (NPDs); and **C**) Common genetic variants from genome-wide association studies (GWAS). For rare variants (A), N Carriers denotes unique individuals analyzed from individual-level data (clinical cohorts and UK Biobank) or aggregated via meta-analysis (meta). HR denotes the Hazard Ratio for developing specific psychiatric disorders (ADHD, BD, MDD) derived from the iPSYCH2015 case-cohort study (Vaez et al., 2024 ^2^, or Sanchez et al., 2023 ^1^). OR denotes the Odds Ratio for ASD or SCZ from Modenato et al., 2021 ^21^ or Kumar et al., 2023 ^41^. N Controls refers to the shared control sets used for CNV (n=782) and SCA (n=870) comparisons. For NPDs (B), values represent aggregated demographics from ENIGMA working groups for individuals with quality-controlled cortical thickness and surface area data. Medication denotes the availability of medication status for subgroup analyses; Subgroups indicate the age cohorts available (Ped: Pediatric, Young: Young Adult, Adult). For common variants (C), sample sizes refer to the discovery GWAS for the psychiatric disorder orcortical metrics. In addition, for polygenic risk score (PRS) analysis, we used CT and SA metrics for 31,413 participants of European (White-British) ancestry from the UK Biobank.

Abbreviations: Abbreviations: ADHD=attention deficit hyperactivity disorder; ASD=autism spectrum disorder; BD=bipolar disorder; CD=conduct disorder; CHR-PS=clinical high risk for psychosis; CNV=copy number variant; Del=deletion; Dup=duplication; ENIGMA=Enhancing Neuro Imaging Genetics through Meta Analysis; MDD=major depressive disorder; NPD=neurodevelopmental and psychiatric disorders; OCD=obsessive-compulsive disorder; PGC=Psychiatric Genomics Consortium; prox.=proximal; SCA=sex chromosome aneuploidy; SCZ=schizophrenia; TS=Turner syndrome; VCFS=Velo-Cardio-Facial syndrome; WBS=Williams-Beuren syndrome.

## Data Availability

UK Biobank data was downloaded under the application 40980 and may be accessed via their standard data access procedure (see http://www.ukbiobank.ac.uk/register-apply). UK Biobank CNVs were called using the pipeline developed in the Jacquemont Lab, as described at https://github.com/MartineauJeanLouis/MIND-GENESPARALLELCNV. The final CNV calls are available for download from the UK Biobank returned datasets (Return ID: 3104, https://biobank.ndph.ox.ac.uk/ukb/dset.cgi?id=3104). The 22q11.2 UCLA raw data are currently available by request from the project PI. Raw neuroimaging data for rare variants are available through request and data access agreement from the PIs of the projects (Brain Canada: S.J. CHUSJ Montreal; 22q11.2: C.E.B. UCLA, Cardiff: D.E.J.L., M.J.O., M.V.B., J.H, Cardiff University; SCA: A.R. NIMH). References to the processing pipeline and R package versions used for analysis are listed in the methods. The GWAS summary statistics are publicly available and can be accessed following the reference papers.

## Code availability

The code for generating all the figures, along with processed summary measures, is available in the following GitHub repository: https://github.com/kkumar-iitkgp/ct_sa_across_disorders_and_variants.git

## References

[1] Sánchez X. C. Associations of psychiatric disorders with sex chromosome aneuploidies in the Danish iPSYCH2015 dataset: a case-cohort study. Lancet Psychiatry 10, 129–138 (2023). 36697121 10.1016/S2215-0366(23)00004-4 PMC9976199

[2] Vaez M. Population-Based Risk of Psychiatric Disorders Associated With Recurrent Copy Number Variants. JAMA Psychiatry (2024) doi:10.1001/jamapsychiatry.2024.1453. PMC11209205 38922630

[3] Jacquemont S. Genes To Mental Health (G2MH): A framework to map the combined effects of rare and common variants on dimensions of cognition and psychopathology. Am. J. Psychiatry (2021). 10.1176/appi.ajp.2021.21040432 PMC9345000 35236119

[4] Owen M. J., Bray N. J., Walters J. T. R. & O’Donovan M. C. Genomics of schizophrenia, bipolar disorder and major depressive disorder. Nat. Rev. Genet. 1–16 (2025). 40355602 10.1038/s41576-025-00843-0

[5] Trubetskoy V. Mapping genomic loci implicates genes and synaptic biology in schizophrenia. Nature 1–13 (2022). 10.1038/s41586-022-04434-5 PMC9392466 35396580

[6] O’Connell K. S. Genomics yields biological and phenotypic insights into bipolar disorder. Nature 1–12 (2025). 10.1038/s41586-024-08468-9 PMC12163093 39843750

[7] Genome-wide analyses of ADHD identify 27 risk loci, refine the genetic architecture and implicate several cognitive domains. Nature https://www.nature.com/articles/s41588-022-01285-8. 10.1038/s41588-022-01285-8 PMC10914347 36702997

[8] Meng X. Multi-ancestry genome-wide association study of major depression aids locus discovery, fine mapping, gene prioritization and causal inference. Nat. Genet. 56, 222–233 (2024). 38177345 10.1038/s41588-023-01596-4 PMC10864182

[9] Brainstorm Consortium Analysis of shared heritability in common disorders of the brain. Science 360, (2018). 10.1126/science.aap8757 PMC6097237 29930110

[10] Grotzinger A. D. The landscape of shared and divergent genetic influences across 14 psychiatric disorders. medRxiv 2025.01.14.25320574 (2025) doi:10.1101/2025.01.14.25320574.

[11] Grotzinger A. D. Mapping the genetic landscape across 14 psychiatric disorders. Nature 1–15 (2025). 10.1038/s41586-025-09820-3 PMC12779569 41372416

[12] Thompson P. M. ENIGMA and global neuroscience: A decade of large-scale studies of the brain in health and disease across more than 40 countries. Transl. Psychiatry 10, 100 (2020). 32198361 10.1038/s41398-020-0705-1 PMC7083923

[13] Boedhoe P. S. W. Subcortical Brain Volume, Regional Cortical Thickness, and Cortical Surface Area Across Disorders: Findings From the ENIGMA ADHD, ASD, and OCD Working Groups. Am. J. Psychiatry 177, 834–843 (2020). 32539527 10.1176/appi.ajp.2020.19030331 PMC8296070

[14] Hibar D. P. Cortical abnormalities in bipolar disorder: an MRI analysis of 6503 individuals from the ENIGMA Bipolar Disorder Working Group. Mol. Psychiatry 23, 932–942 (2018). 28461699 10.1038/mp.2017.73 PMC5668195

[15] Schmaal L. Cortical abnormalities in adults and adolescents with major depression based on brain scans from 20 cohorts worldwide in the ENIGMA Major Depressive Disorder Working Group. Mol. Psychiatry 22, 900–909 (2017). 27137745 10.1038/mp.2016.60 PMC5444023

[16] van Erp T. G. M. Cortical Brain Abnormalities in 4474 Individuals With Schizophrenia and 5098 Control Subjects via the Enhancing Neuro Imaging Genetics Through Meta Analysis (ENIGMA) Consortium. Biol. Psychiatry 84, 644–654 (2018). 29960671 10.1016/j.biopsych.2018.04.023 PMC6177304

[17] Patel Y. Virtual histology of cortical thickness and shared neurobiology in 6 psychiatric disorders. JAMA Psychiatry 78, 47–63 (2021). 32857118 10.1001/jamapsychiatry.2020.2694 PMC7450410

[18] Hettwer M. D. Coordinated cortical thickness alterations across six neurodevelopmental and psychiatric disorders. Nat. Commun. 13, 6851 (2022). 36369423 10.1038/s41467-022-34367-6 PMC9652311

[19] Cao Z. Cortical profiles of numerous psychiatric disorders and normal development share a common pattern. Mol. Psychiatry (2022) doi:10.1038/s41380-022-01855-6. PMC12659924 36380235

[20] Patel Y. Virtual Ontogeny of Cortical Growth Preceding Mental Illness. Biol. Psychiatry (2022) doi:10.1016/j.biopsych.2022.02.959. PMC11080987 35489875

[21] Modenato C. Lessons learnt from neuroimaging studies of Copy Number Variants, a systematic review. Biol. Psychiatry (2021) doi:10.1016/j.biopsych.2021.05.028. 34509290

[22] Modenato C. Effects of eight neuropsychiatric copy number variants on human brain structure. Transl. Psychiatry 11, 399 (2021). 34285187 10.1038/s41398-021-01490-9 PMC8292542

[23] Sønderby I. E. Effects of copy number variations on brain structure and risk for psychiatric illness: Large-scale studies from the ENIGMA working groups on CNVs. Hum. Brain Mapp. 43, 300–328 (2021). 33615640 10.1002/hbm.25354 PMC8675420

[24] Grasby K. L. The genetic architecture of the human cerebral cortex. Science 367, (2020). 10.1126/science.aay6690 PMC7295264 32193296

[25] Makowski C. Discovery of genomic loci of the human cerebral cortex using genetically informed brain atlases. Science 375, 522–528 (2022). 35113692 10.1126/science.abe8457 PMC9469470

[26] Bethlehem R. A. I. Brain charts for the human lifespan. Nature 604, 525–533 (2022). 35388223 10.1038/s41586-022-04554-y PMC9021021

[27] Paus T. Population Neuroscience: Principles and advances. Curr. Top. Behav. Neurosci. 68, 3–34 (2024). 38589637 10.1007/7854_2024_474

[28] Rakic P. Specification of cerebral cortical areas. Science 241, 170–176 (1988). 3291116 10.1126/science.3291116

[29] Sha Z. The overlapping genetic architecture of psychiatric disorders and cortical brain structure. Nat. Ment. Health 1–17 (2025). 10.1038/s44220-025-00475-7 PMC13332534 42404379

[30] Stauffer E.-M. The genetic relationships between brain structure and schizophrenia. Nat. Commun. 14, 7820 (2023). 38016951 10.1038/s41467-023-43567-7 PMC10684873

[31] Cheng W. Genetic Association Between Schizophrenia and Cortical Brain Surface Area and Thickness. JAMA Psychiatry (2021) doi:10.1001/jamapsychiatry.2021.1435. PMC8223140 34160554

[32] Berry A. S. F. A genome-first study of sex chromosome aneuploidies provides evidence of Y chromosome dosage effects on autism risk. Nat. Commun. 15, 8897 (2024). 39406744 10.1038/s41467-024-53211-7 PMC11480344

[33] Raznahan A., Won H., Glahn D. C. & Jacquemont S. Convergence and Divergence of Rare Genetic Disorders on Brain Phenotypes: A Review. JAMA Psychiatry (2022) doi:10.1001/jamapsychiatry.2022.1450. 35767289

[34] Levitis E. The variegation of human brain vulnerability to rare genetic disorders and convergence with behaviorally defined disorders. Biol. Psychiatry (2023) doi:10.1016/j.biopsych.2023.07.008. PMC10799187 37480975

[35] Mollon J., Almasy L., Jacquemont S. & Glahn D. C. The contribution of copy number variants to psychiatric symptoms and cognitive ability. Mol. Psychiatry 28, 1480–1493 (2023). 36737482 10.1038/s41380-023-01978-4 PMC10213133

[36] Zimmerman M., Morgan T. A. & Stanton K. The severity of psychiatric disorders: World Psychiatry. World Psychiatry 17, 258–275 (2018). 30192110 10.1002/wps.20569 PMC6127765

[37] de Zwarte S. M. C. The association between familial risk and brain abnormalities is disease specific: An ENIGMA-relatives study of schizophrenia and bipolar disorder. Biol. Psychiatry 86, 545–556 (2019). 31443932 10.1016/j.biopsych.2019.03.985 PMC7068800

[38] Opel N. Cross-Disorder Analysis of Brain Structural Abnormalities in Six Major Psychiatric Disorders: A Secondary Analysis of Mega- and Meta-analytical Findings From the ENIGMA Consortium. Biol. Psychiatry 88, 678–686 (2020). 32646651 10.1016/j.biopsych.2020.04.027

[39] Sydnor V. J. Neurodevelopment of the association cortices: Patterns, mechanisms, and implications for psychopathology. Neuron Preprint at 10.1016/j.neuron.2021.06.016 (2021). PMC8448958 34270921

[40] Markello R. D. neuromaps: structural and functional interpretation of brain maps. Nat. Methods 19, 1472–1479 (2022). 36203018 10.1038/s41592-022-01625-w PMC9636018

[41] Kumar K. Subcortical Brain Alterations in Carriers of Genomic Copy Number Variants. Am. J. Psychiatry 180, 685–698 (2023). 37434504 10.1176/appi.ajp.20220304 PMC10885337

[42] Norbom L. B. New insights into the dynamic development of the cerebral cortex in childhood and adolescence: Integrating macro- and microstructural MRI findings. Prog. Neurobiol. 204, 102109 (2021). 34147583 10.1016/j.pneurobio.2021.102109

[43] Liao Z. Copy number variants and the tangential expansion of the cerebral cortex. Nat. Commun. 16, 1–12 (2025). 39962045 10.1038/s41467-025-56855-1 PMC11833094

[44] Voineskos A. N. Effects of antipsychotic medication on brain structure in patients with major depressive disorder and psychotic features: Neuroimaging findings in the context of a randomized placebo-controlled clinical trial: Neuroimaging findings in the context of a randomized placebo-controlled clinical trial. JAMA Psychiatry 77, 674–683 (2020). 32101271 10.1001/jamapsychiatry.2020.0036 PMC7330722

[45] Sun D. Large-scale mapping of cortical alterations in 22q11.2 deletion syndrome: Convergence with idiopathic psychosis and effects of deletion size. Mol. Psychiatry 25, 1822–1834 (2020). 29895892 10.1038/s41380-018-0078-5 PMC6292748

[46] Hindley G. Charting the landscape of genetic overlap between mental disorders and related traits beyond genetic correlation. Am. J. Psychiatry 179, 833–843 (2022). 36069018 10.1176/appi.ajp.21101051 PMC9633354

[47] Kazem S. Determinants of pleiotropy and monotonic gene dosage responses across human traits. Genetic and Genomic Medicine (2025).

[48] Auwerx C., Moix S., Kutalik Z. & Reymond A. Disentangling mechanisms behind the pleiotropic effects of proximal 16p11.2 BP4–5 CNVs. Am. J. Hum. Genet. 111, 2347–2361 (2024). 39332408 10.1016/j.ajhg.2024.08.014 PMC11568757

[49] Sudlow C. UK biobank: an open access resource for identifying the causes of a wide range of complex diseases of middle and old age. PLoS Med. 12, e1001779 (2015). 25826379 10.1371/journal.pmed.1001779 PMC4380465

[50] Hoogman M. Brain Imaging of the Cortex in ADHD: A Coordinated Analysis of Large-Scale Clinical and Population-Based Samples. Am. J. Psychiatry 176, 531–542 (2019). 31014101 10.1176/appi.ajp.2019.18091033 PMC6879185

[51] van Rooij D. Cortical and subcortical brain morphometry differences between patients with autism spectrum disorder and healthy individuals across the lifespan: Results from the ENIGMA ASD working group. Am. J. Psychiatry 175, 359–369 (2018). 29145754 10.1176/appi.ajp.2017.17010100 PMC6546164

[52] ENIGMA Clinical High Risk for Psychosis Working Group Association of structural magnetic resonance imaging measures with psychosis onset in individuals at Clinical High Risk for developing psychosis: An ENIGMA working group mega-analysis: An ENIGMA working group mega-analysis. JAMA Psychiatry 78, 753–766 (2021). 33950164 10.1001/jamapsychiatry.2021.0638 PMC8100913

[53] Gao Y., Staginnus M. & ENIGMA-Antisocial Behavior Working Group. Cortical structure and subcortical volumes in conduct disorder: a coordinated analysis of 15 international cohorts from the ENIGMA-Antisocial Behavior Working Group. Lancet Psychiatry 11, 620–632 (2024). 39025633 10.1016/S2215-0366(24)00187-1

[54] Boedhoe P. S. W. Cortical abnormalities associated with pediatric and adult obsessive-compulsive disorder: Findings from the ENIGMA obsessive-compulsive disorder working group. Am. J. Psychiatry 175, 453–462 (2018). 29377733 10.1176/appi.ajp.2017.17050485 PMC7106947

[55] Fischl B. Automatically parcellating the human cerebral cortex. Cereb. Cortex 14, 11–22 (2004). 14654453 10.1093/cercor/bhg087

[56] Alfaro-Almagro F. Image processing and Quality Control for the first 10,000 brain imaging datasets from UK Biobank. Neuroimage 166, 400–424 (2018). 29079522 10.1016/j.neuroimage.2017.10.034 PMC5770339

[57] Lê S., Josse J. & Husson F. FactoMineR: AnRPackage for Multivariate Analysis. J. Stat. Softw. 25, 1–18 (2008).

[58] Alexander-Bloch A. F. On testing for spatial correspondence between maps of human brain structure and function. Neuroimage 178, 540–551 (2018). 29860082 10.1016/j.neuroimage.2018.05.070 PMC6095687

[59] Mowinckel A. M. & Vidal-Piñeiro D. Visualization of brain statistics with R packages ggseg and ggseg3d. Adv. Methods Pract. Psychol. Sci. 3, 466–483 (2020).

